# Optimal seamline detection for SAR image mosaicking guided by superpixel segmentation and region merging

**DOI:** 10.1371/journal.pone.0348842

**Published:** 2026-05-08

**Authors:** Dong Yan, Feixiang Zeng, Bairu Chen, Rui Huang, Yi She

**Affiliations:** 1 Spatiotemporal Information Center, Powerchina Guiyang Engineering Corporation Limited, Guiyang, Guizhou, China; 2 School of Geosciences and Info-Physics, Central South University, Changsha, Hunan, China; 3 Ocean College, Zhejiang University, Zhoushan, Zhejiang, China; Leibniz University Hannover, GERMANY

## Abstract

Large-scale studies and applications of SAR images require the mosaicking of multiple scenes. However, geometric misregistration and radiometric inconsistencies among adjacent images often lead to poor continuity and unnatural transitions in the mosaicked images, which severely restrict the effectiveness of SAR images in large-area information analysis and retrieval. Optimal seamline detection seeks to determine the most suitable stitching path within the overlapping regions of adjacent images, ensuring that mosaicked SAR images exhibit maximal consistency in intensity, texture, and geometric features while minimizing stitching artifacts and visual discontinuities. Existing seamline detection methods, however, are often limited by their obstacle-avoidance capability and computational efficiency. To overcome these limitations, this study proposes an optimal seamline detection approach for SAR images guided by superpixel segmentation and region merging. First, the Patch-Based SLIC (PB-SLIC) algorithm is enhanced to achieve consistent superpixel segmentation across multiple overlapping images. Second, a region adjacency graph is constructed by integrating Bhattacharyya distance, texture distribution, and boundary length information, which guides the merging of superpixels and produces candidate seamlines that preserve object integrity and accurately follow object boundaries. Then, an initial seamline network is generated using area Voronoi diagrams with overlap (AVDO), and a cost function based on normalized cross-correlation is established. The seamline network is further refined using a shortest-path algorithm to extract the optimal seamline network from the candidates. Finally, using real SAR datasets, we analyze and verify the effectiveness of superpixel segmentation and region merging in seamline detection. Comparative experiments with two classical methods further demonstrate that the proposed approach achieves superior obstacle-avoidance capability and shorter search time, while ensuring higher mosaicking quality and significantly improving computational efficiency.

## 1 Introduction

Synthetic Aperture Radar (SAR) is an active microwave remote sensing technology that supports all-weather, day-and-night ground observation by transmitting penetrating electromagnetic waves and receiving their backscattered signals, thereby overcoming the weather and illumination constraints of traditional optical remote sensing. It has been widely employed in disaster assessment, environmental monitoring, and resource exploration [1–3]. The increasing number and extended operational duration of SAR satellites have generated massive datasets, providing a foundation for large-area SAR image research and applications. However, due to the limitations of SAR sensor design, a single SAR image often cannot fully cover the entire study area. Therefore, it is necessary to mosaic multiple SAR images to obtain seamless composite images.

Ideally, images acquired by the same sensor maintain good geometric consistency and can be directly mosaicked into large-scale SAR image with satisfactory visual quality. In practice, however, multiple orthoimages covering the same area are often captured at different times and from different orbits, inevitably influenced by surface changes, variations in incidence angles, and sensor attitude differences. These factors cause significant geometric misalignments between adjacent images, severely reducing both the visual quality of the mosaicked images and the accuracy of information extraction. Optimal seamline detection aims to identify the best stitching path within overlapping regions of adjacent images. This path should avoid crossing through complete objects while preferentially traversing regions with minimal differences. Mosaicking along this path can effectively mitigate geometric misalignments between images. The objective of this study is to develop an efficient and high-quality optimal seamline network to eliminate visual artifacts and enhance the visual quality of mosaicked images.

The essence of optimal seamline detection is to construct a cost function based on intensity, texture, gradient, and other information in the overlapping regions of images, and then identifying the optimal path by minimizing the total cost using optimization algorithms. For example, the bottleneck model [4] minimizes a cost function related to intensity differences, enabling the seamline to avoid regions with large intensity variations and effectively mosaicking images with simple textures. The twin-snake model [5] defines the seamline as the path traversed by two “snakes”, which move toward each other from the boundaries of the overlapping region based on an energy function. This model not only considers intensity differences but also incorporates texture differences into the energy function, thereby improving mosaicking performance. Pan et al. [6] proposed a segmentation-based seamline detection method, which first identifies candidate regions through image segmentation, then establishes a cost function based on differences within these regions, and finally applies the shortest-path algorithm to extract the optimal seamline. Similarly, Pang, Yuan and Wang [7–9] obtained candidate regions using Semi-Global Matching (SGM) or watershed segmentation algorithm, and then applied the shortest-path algorithm within these regions to determine pixel-level optimal seamlines while avoiding paths that cross obstacles. In addition, Dynamic Programming (DP) algorithms are also widely used for seamline detection. Shen et al. [10] constructed a cost function based on intensity difference, gradient similarity, and geometric disparity, and subsequently employed DP to search for the optimal seamline within the overlapping region of adjacent images. To enhance obstacle-avoidance capability, Li et al. [11] proposed the Automatic Piecewise Dynamic Programming (APDP) algorithm, which divides the overlapping region into multiple consecutive segments. Within each segment, the algorithm searches along five directions to find the path with the lowest average cost, and then connects the optimal paths from all segments to obtain a globally optimal seamline. However, when applied to large datasets, such methods often rely on sequential mosaicking, selecting one image as the base and recursively merging others, which leads to high computational cost and strong dependence on mosaicking order, thereby limiting both efficiency and quality.

To further improve mosaicking efficiency, Hsu et al. [12] generated Voronoi diagrams (VD) using image centers as seed points and employed the shared Voronoi edges as seamlines for images with large overlaps. Subsequently, to handle small-overlap cases, Pan et al. [13,14] proposed AVDO to automatically generate an initial seamline network, which they subsequently refined using bottleneck or shortest-path algorithms, thereby enabling simultaneous mosaicking of all images. Yuan et al. [15] proposed a seamline network generation method based on the medial axis of a Voronoi diagram, effectively addressing the issue of stitching gaps commonly encountered in such approaches. Hong et al. [16] and Zhang et al. [17] generated initial seamlines using Voronoi diagrams and constructed buffer zones along these initial seamlines. Subsequently, they employed the A* algorithm and the shortest-path algorithm, respectively, to search for the optimal seamline within the defined buffer regions. For UAV image mosaicking, Song et al. [18] and Wang et al. [19] refined Voronoi-based seamline networks using the watershed segmentation algorithm and the spatial ant colony algorithm, respectively. Peng et al. [20] and Li et al. [21] adopted a registration–search–correction strategy to determine the optimal seamline. Specifically, they first performed high-precision image registration, then integrated spatial and spectral information to construct an energy function or employed the weighted fast sweeping algorithm to extract the seamline. Finally, graph-cut optimization or refined registration techniques were applied to eliminate artifacts in the vicinity of the seamline. These approaches obtain the optimal seamline network via global optimization, eliminating dependence on mosaicking order and greatly improving efficiency through parallel processing. Nonetheless, when refining the initial network, they still require pixel-wise searches in overlapping regions, which can cause seamlines to cross intact objects.

Li et al. [22] applied a superpixel segmentation algorithm to cluster the pixels in overlapping regions of images and, by integrating intensity, gradient, and texture information, formulated an energy function. Using graph-cut algorithm, they extracted the optimal seamline with the minimum cumulative energy. Yuan et al. [23] defined the images to be mosaicked as reference and target, applied superpixel segmentation to the reference image within the overlap, and then extracted the seamline using graph cuts. Liu et al. [24] employed the fractal net evolution approach (FNEA) to jointly segment overlapping regions of SAR images, overlapping, and finally applied the shortest-path algorithm to identify the minimum-cost seamline. Wang et al. [25] first generated direct seamlines for UAV images, and subsequently constructed an energy function incorporating color, texture, and gradient features to perform superpixel segmentation. Finally, a multilabel optimization algorithm was employed to extract the optimal seamline from the resulting segmentation boundaries. These methods aggregate adjacent pixels with similar features into compact and continuous regions, which not only reduces the computational cost of image processing but also improves the preservation of object contours. Therefore, incorporating superpixel segmentation can effectively improve the quality and efficiency of optimal seamline detection. However, obtaining consistent segmentation results in overlapping regions is a prerequisite for these methods. The most straightforward strategy is to segment one image in the overlap and project the result onto the others [22,23]. Yet, due to surface changes and other factors, the segmentation may not match the other images. Hence, all images should be jointly considered during segmentation. In change detection and target detection, difference maps [26,27] or fusion maps [28,29] generated from overlapping regions of two images are typically used to achieve consistent segmentation. Nevertheless, such methods are limited to pairwise overlaps, do not directly segment the original images, and thus cannot guarantee accuracy and stability when more than two images overlap [30]. Furthermore, while deliberate over-segmentation is often employed to capture object boundaries more precisely, it leads to object fragmentation that adversely affects seamline detection and reduces seamline quality.

To address these challenges, this study improves the PB-SLIC algorithm [31] by incorporating comprehensive information from overlapping regions, enabling accurate, stable, and consistent superpixel segmentation across multiple SAR images. Based on this, a region adjacency graph (RAG) is constructed with superpixels as nodes, and adjacent superpixels with similar characteristics are merged using a region-merging algorithm that integrates Bhattacharyya distance, texture features, and boundary length. This process mitigates the adverse effects of over-segmentation while generating candidate seamlines that both preserve object integrity and accurately follow object boundaries. Subsequently, an initial seamline network is generated using AVDO. A normalized cross-correlation coefficient, computed from fixed-window pixel information, is adopted as the cost function to adjust network nodes so that they align with the candidate seamlines. Finally, pixels along these candidate seamlines are defined as valid, and Dijkstra’s algorithm is applied to extract the minimum-cost connections between adjacent nodes, thereby forming the optimal seamlines. All optimal seamlines are then automatically integrated to construct the complete seamline network. The main contributions of this study are as follows:

1. A novel superpixel segmentation method tailored for multiple SAR images is developed based on patch similarity, enabling the direct generation of consistent segmentation results within overlapping regions.
2. We further construct a region adjacency graph by integrating multiple similarity measures and develop a region-merging strategy to address over-segmentation and fragmented objects. The resulting candidate seamlines effectively avoid traversing intact objects while accurately capturing object boundaries.
3. A robust cost function is designed by comprehensively exploiting pixel information within a fixed window. This cost function reduces the dependency on image registration accuracy while enhancing the stability of seamline optimization.

In summary, building on a comprehensive review of existing studies, this study proposes an optimal seamline detection method for SAR image mosaicking guided by superpixel segmentation and region merging, with the goal of mitigating geometric misalignments in mosaicked SAR images. The proposed method effectively enhances both the quality and efficiency of optimal seamline network construction.

## 2 Materials and methods

### 2.1 Overview

**Fig 1:**
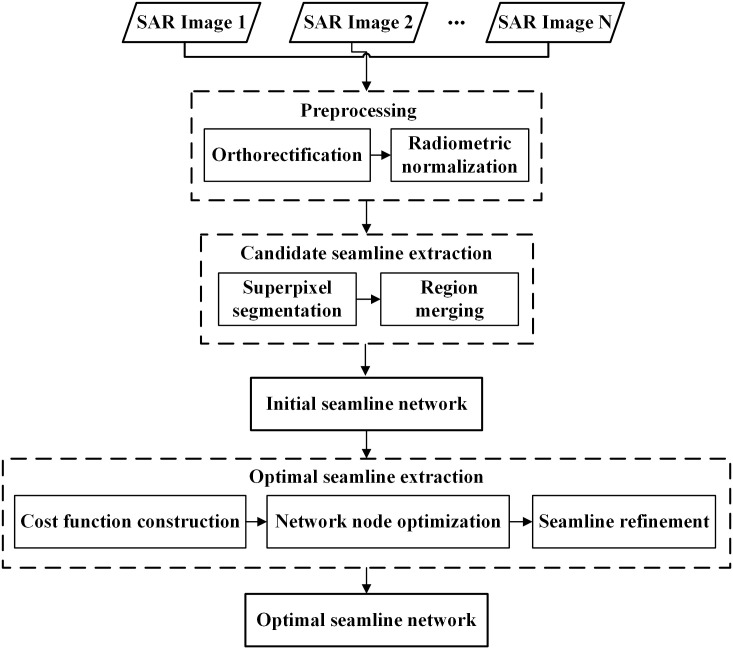
Framework diagram of the proposed method.

As illustrated in Fig 1, prior to seamline detection, multiple SAR images covering the study area are first subjected to orthorectification and radiometric normalization to generate radiometrically consistent orthoimages. Given the challenges of obtaining accurate and stable consistent segmentation in overlapping regions using traditional difference or fusion maps, this study leverages difference information from multiple SAR images in overlapping areas. By enhancing the PB-SLIC algorithm, consistent superpixel segmentation of multiple SAR images within overlapping regions is achieved. To further address over-segmentation and improve the quality of candidate seamlines, a RAG is constructed based on multiple similarity measures to guide superpixel merging, thereby extracting high-quality candidate seamlines. Building on this, a cost function independent of image registration accuracy is formulated to optimize initial network nodes, and the shortest-path algorithm is applied to extract the optimal seamlines, ultimately forming the final seamline network.

### 2.2 Candidate seamline extraction

#### 2.2.1 Consistent superpixel segmentation in overlapping regions.

Superpixel segmentation partitions an image into multiple non-overlapping and connected regions, in which pixels within the same region exhibit strong homogeneity in the feature space or statistical characteristics, while adjacent regions demonstrate pronounced heterogeneity. During mosaicking, the seamline should be distributed as much as possible along object boundaries and avoid traversing homogeneous regions, so as to preserve the structural integrity and continuity of ground objects. Since the boundaries between neighboring superpixels are highly consistent with actual object boundaries, a close spatial correspondence is established between them. Therefore, the superpixel boundaries can serve as an effective constraint to guide the search for the optimal seamline.

Simple Linear Iterative Clustering (SLIC) [32] clusters pixels iteratively within a localized region based on color similarity and spatial proximity, thereby generating uniform and coherent superpixels. Due to the abundant color information of optical images in the [l, a, b] space, SLIC is particularly well-suited for optical image. In contrast, single-channel SAR images contain only intensity information and are significantly affected by speckle noise. When similarity is measured solely by intensity differences, direct application of SLIC often yields unsatisfactory segmentation results. SAR image patches contain rich texture and structural cues that can be exploited to assess pixel similarity. Building on this idea, Yu et al. [31] proposed the PB-SLIC method, in which patch similarity is defined as:


$$\delta (\mu_{i},\mu_{j})=2K\cdot \log\left(\frac{{\bar{I}}_{\mu_{i}\cup \mu_{j}}}{\sqrt{{\bar{I}}_{\mu_{i}}\;{\bar{I}}_{\mu_{j}}}}\right)$$ (1)

where $\mu_{i}$ and $\mu_{j}$ denote the patches centered at pixels *i* and *j*, respectively. ${\bar{I}}_{\mu_{i}}$ and ${\bar{I}}_{\mu_{j}}$ represent the mean intensities of patches *i* and *j*, respectively. ${\bar{I}}_{\mu_{i}\cup \mu_{j}}$ is the mean intensity of all pixels in patches *i* and *j*. *K* is the number of pixels in either patch *i* or patch *j*. The patch size is typically set to 5×5.

In addition to patch similarity, the spatial distance between pixels also contributes to their overall similarity, which is consistent with the original SLIC:


$$d(i,j)=\sqrt{(x_{i}-x_{j}{)}^{2}+(y_{i}-y_{j}{)}^{2}}$$ (2)

Finally, the similarity between pixels *i* and *j* is defined as:


$$D(i,j)=\delta (\mu_{i},\mu_{j})+\lambda d(i,j)$$ (3)

where λ is the distance weight that balances the relative importance of spatial proximity and patch similarity. A larger λ produces more regular and compact superpixels, while a smaller λ allows the segmentation results to better adhere to object boundaries.

Although superpixel segmentation has been widely applied in SAR image change detection [33], target detection [34], and image classification [35], the requirements for superpixels differ between applications. The goal of this study is to achieve accurate and consistent segmentation within overlapping regions of SAR images, a task analogous to multi-temporal SAR segmentation. Conventional methods relying on difference maps or fused images do not directly segment the originals, making it difficult to guarantee segmentation accuracy. To overcome this, the PB-SLIC algorithm is improved, and a segmentation method tailored for overlapping regions of multiple SAR images is proposed. This approach enables accurate and consistent segmentation in overlapping regions without relying on difference or fusion processing.

**Fig 2:**
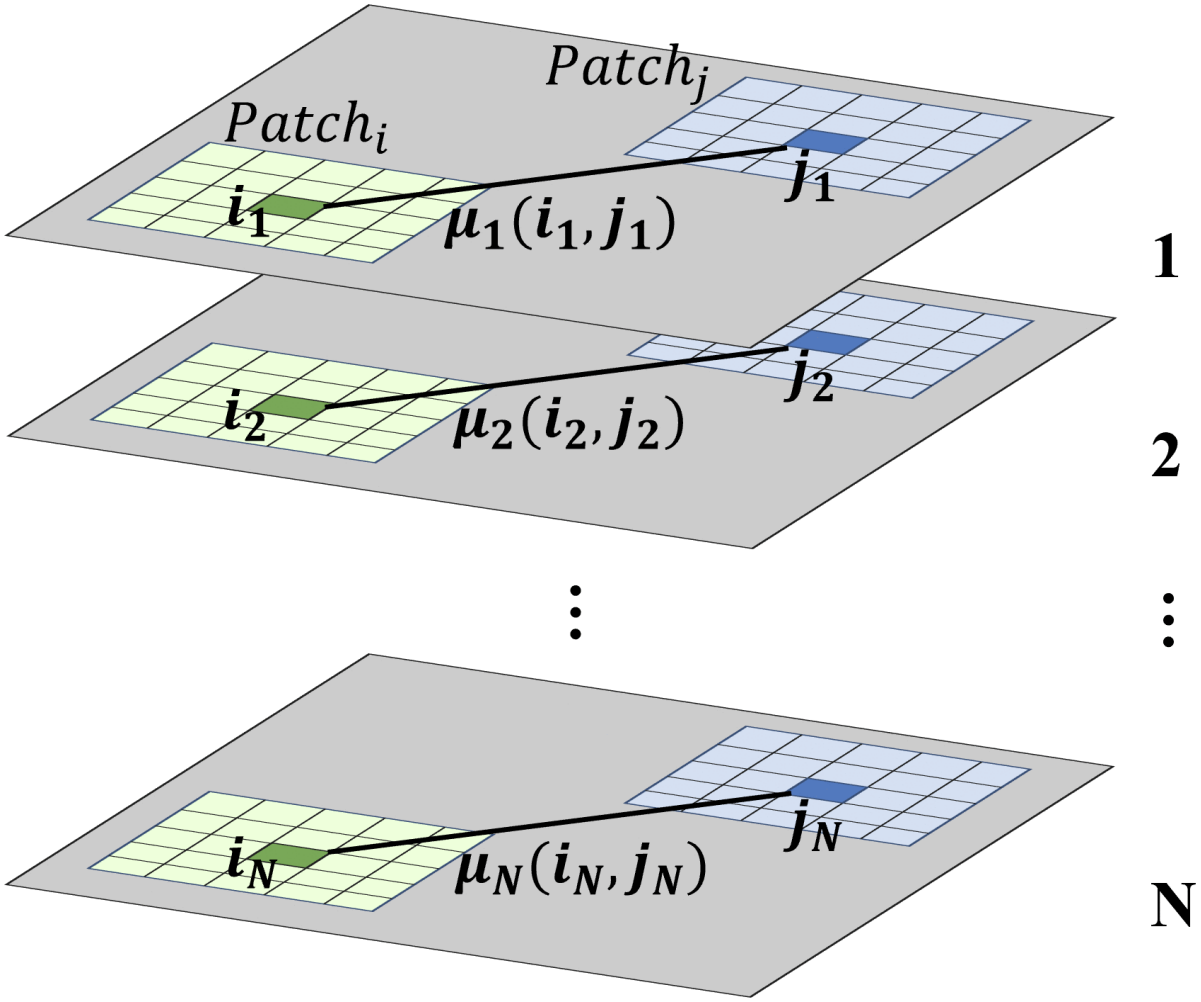
Illustration of intensity differences in overlapping regions.

As illustrated in Fig 2, suppose *M* SAR images overlap the study area, and let any overlapping location be covered by *N* images (N≥1, *N* = 1 indicates no overlap). For any two pixels $i_{n}$ and $j_{n}$ in the *n*-th overlapping image, their patch similarity is defined as:


$$\mu_{n}(i_{n},j_{n})=A\cdot \log\left(\frac{{\bar{I}}_{P_{i}\cup P_{j}}^{n}}{\sqrt{{\bar{I}}_{P_{i}}^{n}\cdot {\bar{I}}_{P_{j}}^{n}}}\right)$$ (4)

where *n* denotes the index of the overlapping image (n=1,2,…,N). $\mu_{n}(i_{n},j_{n})$ denotes the similarity between pixels $i_{n}$ and $j_{n}$ in the *n*-th overlapping image. ${\bar{I}}_{P_{i}}^{n}$ and ${\bar{I}}_{P_{j}}^{n}$ represent the mean intensity values of the patches centered at pixels $i_{n}$ and $j_{n}$, respectively. ${\bar{I}}_{P_{i}\cup P_{j}}^{n}$ and *A* denote the mean intensity of all pixels within the two patches and the number of pixels in a patch, respectively. The patch size is set to 5×5.

Consistent with the original SLIC formulation, the Euclidean distance $d_{s}(i,j)$ between pixels *i* and *j* is computed as:


$$d_{s}(i,j)=\sqrt{(x_{i}-x_{j}{)}^{2}+(y_{i}-y_{j}{)}^{2}}$$ (5)

where $\left(x_{i},y_{i}\right)$ and $\left(x_{j},y_{j}\right)$ denote the two-dimensional coordinates of pixels *i* and *j*, respectively.

Finally, the similarity between pixels *i* and *j* within the overlapping region is defined as:


$$D(i,j)=\sqrt{\frac{\mu_{1}^{2}(i_{1},j_{1})+\mu_{2}^{2}(i_{2},j_{2})+\cdots +\mu_{N}^{2}(i_{N},j_{N})}{N}}+m\cdot d_{s}(i,j)$$ (6)

where *m* is the compactness factor, which is set to 0.2 in this study.

Following the SLIC framework, the overlapping region is segmented as follows: (1) identify overlapping regions across images and initialize cluster centers on an S×S grid; (2) iteratively update cluster centers for 10 iterations by associating pixels within a 2S×2S neighborhood according to Eq (6); (3) assign isolated pixels to the nearest superpixel.

#### 2.2.2 Superpixel region merging with multiple similarity measures.

To ensure that optimal seamlines closely adhere to object boundaries, SAR images are often deliberately over-segmented. However, this over-segmentation may degrade seamline quality. To address this, a region-merging algorithm is employed to recombine the initial superpixels generated in the previous step, thereby mitigating the adverse effects of over-segmentation. By iteratively merging superpixel pairs whose merging cost is below a prescribed threshold, over-segmentation can be effectively reduced, yielding segmentation outputs that better conform to true object boundaries. Typically, the merging cost is defined according to the similarity between adjacent superpixels. An ineffective similarity measure may leave regions that should be merged separate, or conversely merge regions that should remain distinct. Therefore, accurate quantification of inter-superpixel similarity is critical for high-quality merging. Fan et al. [36] replaced the mean-based statistical similarity measure (SSM) with the Bhattacharyya Distance (BD), and integrated it with a texture pattern similarity measure (TPSM) and a relative common boundary length penalty (RCBLP) to form an effective superpixel similarity metric for SAR image.

A SSM can be constructed using the statistics of pixels within two regions to determine whether they belong to homogeneous regions. Unlike optical images, where similarity is often based on differences in pixel means or grayscale distributions, SAR images are strongly affected by multiplicative speckle noise. Hence, similarity is often defined based on the ratio of mean intensities within regions [37]:


$$\text{SSM}(\Omega_{1},\Omega_{2})=\frac{\sqrt{L}\left[1-\min\left(\frac{{\bar{m}}_{1}}{{\bar{m}}_{2}},\frac{{\bar{m}}_{2}}{{\bar{m}}_{1}}\right)\right]}{\sqrt{\frac{1}{n_{1}}{\left[\min\left(\frac{{\bar{m}}_{1}}{{\bar{m}}_{2}},1\right)\right]}^{2}+\frac{1}{n_{2}}{\left[\min\left(\frac{{\bar{m}}_{2}}{{\bar{m}}_{1}},1\right)\right]}^{2}}}$$ (7)

where $\text{SSM}(\Omega_{1},\Omega_{2})$ represents the statistical similarity between superpixels 1 and 2. *L* is the number of looks in the SAR intensity image. ${\bar{m}}_{1}$ and ${\bar{m}}_{2}$ are the mean intensities of superpixels 1 and 2, respectively, and *n*_1_, *n*_2_ are the corresponding pixel counts.

Although mean-based SSM can distinguish differences in homogeneous regions, it performs poorly in heterogeneous areas [36]. To obtain robust merging in such regions, the Bhattacharyya Distance (BD) is used as an alternative statistical metric:


$$\text{BD}(\Omega_{1},\Omega_{2})=-\ln\sum_{i=1}^{Q}\sqrt{h_{\Omega_{1}}(i)\;h_{\Omega_{2}}(i)}$$ (8)

where $\text{BD}(\Omega_{1},\Omega_{2})$ denotes the Bhattacharyya Distance between superpixels 1 and 2. $h_{\Omega_{1}}(i)$ and $h_{\Omega_{2}}(i)$ are the normalized histograms of the superpixels, and *Q* is the number of histogram bins.

SAR images contain rich texture information, and statistical measures alone may not effectively discriminate complex textural differences. Therefore, a reasonable texture similarity measure is introduced. Texture characterizes the spatial order or disorder of intensity variations within a superpixel and can be described using a covariance matrix [38]:


$$R(\Omega )=\frac{1}{n}\sum_{(x,y)\in \Omega }\sum_{-w\leq x_{0},y_{0}\leq w}\left[I(x,y)-{\bar{I}}_{\Omega }\right]\left[I(x+x_{0},y+y_{0})-{\bar{I}}_{\Omega }\right]$$ (9)

where *I*(*x*,*y*) is the pixel intensity at (*x*,*y*), ${\bar{I}}_{\Omega }$ is the mean intensity within the superpixel, and *w* is the window size.

Using covariance descriptors, the TPSM between two superpixels is defined as:


$$\text{TPSM}(\Omega_{1},\Omega_{2})=\frac{\|R(\Omega_{1})-R(\Omega_{2}){\|}_{2}}{\|R(\Omega_{1}){\|}_{2}+\|R(\Omega_{2}){\|}_{2}}$$ (10)

where $\text{TPSM}(\Omega_{1},\Omega_{2})$ represents the texture pattern similarity measure between superpixels 1 and 2. Smaller values indicate smaller texture differences, and $\|\cdot {\|}_{2}$ denotes the Euclidean (L2) norm.

Additionally, the superpixel merging cost typically consists of two components [39]: (1) a statistical goodness-of-fit (SGOF) term, which increases as merging progresses, and (2) a boundary-related penalty term, which decreases as merging progresses. The BD-based statistical similarity and TPSM only reflect the SGOF, lacking a boundary-related penalty. To encourage minimal boundary lengths in the final merged superpixels, Shui et al. [37] proposed the RCBLP:


$$\text{RCBLP}(\Omega_{1},\Omega_{2})=\frac{\text{Length}(\Omega_{1}\cap \Omega_{2})}{\sqrt{\min(n_{1},n_{2})}}$$ (11)

where $\text{Length}(\Omega_{1}\cap \Omega_{2})$ denotes the number of pixels along the common boundary of the two superpixels.

Finally, the BD-based statistical similarity, TPSM, and RCBLP are combined with weighting to form the final superpixel similarity measure [36]:


$$D(\Omega_{1},\Omega_{2})=\sqrt{\min(n_{1},n_{2})}\;\text{BD}(\Omega_{1},\Omega_{2})+a\frac{{\text{TPSM}}^{2}(\Omega_{1},\Omega_{2})}{0.5+{\text{TPSM}}^{2}(\Omega_{1},\Omega_{2})}-b\;\text{RCBLP}(\Omega_{1},\Omega_{2})$$ (12)

where *a* and *b* are positive weighting coefficients. Parameter *a* can be increased for images with richer texture, while *b* controls the length of merged boundaries. Weighting the BD by superpixel size improves merging reliability in heterogeneous regions.

The region adjacency graph (RAG) is a commonly used graph structure in image processing and computer vision that represents the adjacency relationships between different regions in an image. It has been widely applied in superpixel merging [40], post-processing of image segmentation [41], object detection, and other related tasks. In the RAG, nodes correspond to superpixels, and edges connect adjacent regions with weights equal to their pairwise similarity. This structure effectively combines spatial information and regional features for fine-grained image analysis and processing.

**Fig 3:**
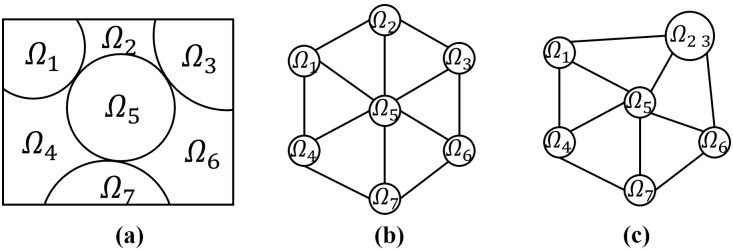
Superpixel merging process based on the RAG. **(a)** Adjacency relationships among superpixels. **(b)** Initial region adjacency graph. **(c)** Region adjacency graph after merging.

In superpixel merging, the initially segmented superpixels are typically used as nodes, and the similarity between superpixels is assigned as edge weights to construct the initial RAG. As illustrated in Fig 3(a), suppose the initially segmented superpixels are $\Omega_{1},\Omega_{2},\ldots ,\Omega_{7}$. If two superpixels are adjacent, their similarity is computed according to Eq (12); otherwise, their similarity is set to infinity:


$$W(i,j)=\left\{ \begin{array}{cc} D(\Omega_{i},\Omega_{j}), & \text{if }\Omega_{i}\text{ and }\Omega_{j}\text{ are adjacent}\\ \infty , & \text{otherwise} \end{array}\right.$$ (13)

The similarity between all superpixels is calculated, and these values are used as edge weights to construct the initial RAG (Fig 3(b)), where smaller weights indicate greater similarity. During merging, the edge with the smallest weight is selected, and the corresponding pair of superpixels is merged. The RAG is then updated by recomputing weights between the merged region and its neighboring regions. For instance, if superpixels $\Omega_{2}$ and $\Omega_{3}$ are most similar, they are merged into $\Omega_{23}$. Subsequently, the similarities between $\Omega_{23}$ and other superpixels are recalculated according to Eq (13), producing a new RAG as illustrated in Fig 3(c). This process iterates until a prescribed number of merges is reached or the minimum finite edge weight exceeds a predefined threshold.

### 2.3 Construction of the Optimal Seamline Network

#### 2.3.1 Construction of the Initial Seamline Network.

In conventional multiple image mosaicking, adjacent images are typically merged sequentially. Two images are first combined into a new image, which is then iteratively merged with other neighboring images until a complete mosaic is obtained. This sequential strategy is inefficient and produces numerous intermediate results, thereby increasing storage demands. To overcome these limitations, this study adopts a global strategy by constructing an optimal seamline network that simultaneously mosaics all images, thus enhancing overall efficiency.

**Fig 4:**
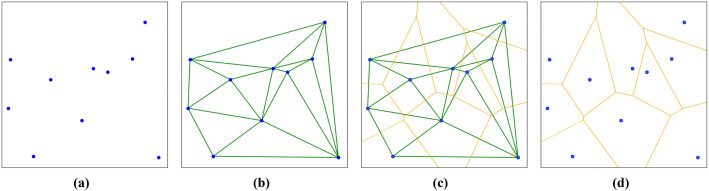
Voronoi diagram construction process. **(a)** Seed points. **(b)** Delaunay triangulation. **(c)** Voronoi diagram construction. **(d)** Voronoi diagram.

The Voronoi diagram (VD) is a mathematical structure that partitions space into regions based on a set of seed points, where each region contains all points closer to its corresponding seed point than to any other [42]. The VD is the dual of the Delaunay triangulation and can therefore be derived from it. The construction process is as follows: (1) generate a Delaunay triangulation from the given seed points, following the maximum–minimum angle criterion to ensure triangle quality; (2) draw perpendicular bisectors for all triangle edges; (3) define the intersections of these bisectors as vertices of the VD; and (4) connect these vertices sequentially to complete the VD. The process is illustrated in Fig 4.

**Fig 5:**
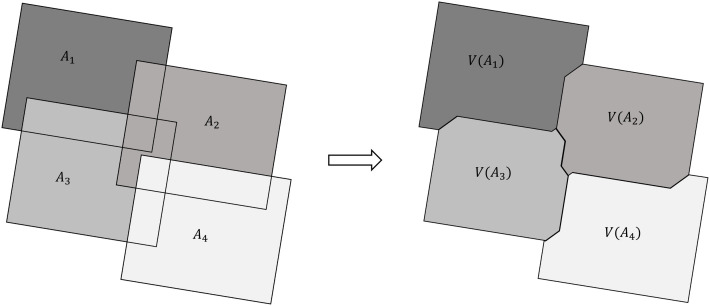
AVDO Illustration.

In conventional VD-based seamline detection algorithms [12], seed points are typically placed at image centers to construct a VD, and the edges of Voronoi cells are used as seamlines. However, this method is valid only when all Voronoi vertices fall within over-lapping regions. If vertices lie outside these areas, erroneous seamlines may be produced [43]. To overcome this limitation, Pan et al. [13] extended the VD from one to two dimensions and proposed an AVDO for seamline detection. The concept is illustrated in Fig 5.

**Fig 6:**
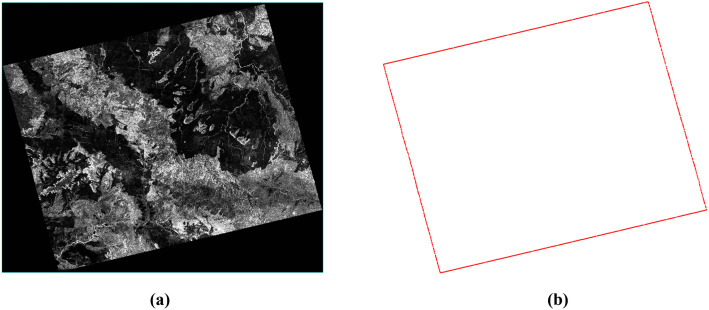
Illustration of Valid Regions. (a) Orthorectified SAR image. **(b)** Contour lines of valid regions.

**Fig 7:**
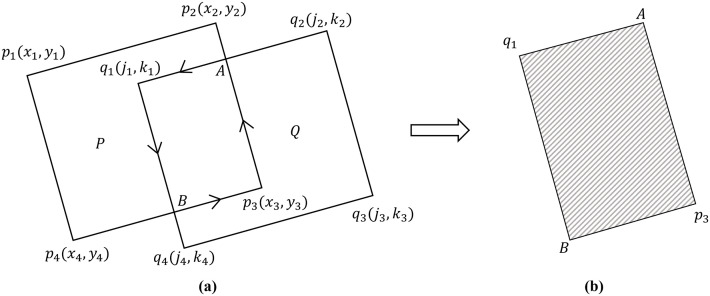
Illustration of Overlapping Area Extraction. **(a)** Searching Process. **(b)** Overlapping Area.

**Fig 8:**
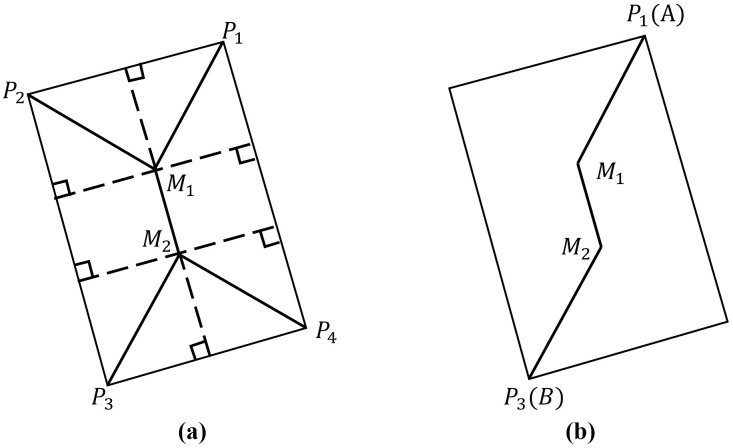
Skeleton Lines of Overlapping Regions.

When generating the initial seamline network using AVDO, the seamlines consist of the shared edges of the Voronoi cells. By definition, these shared edges correspond to the skeletons or medial axes of the overlapping regions between adjacent images. The extraction of skeletons from overlapping regions involves the following steps:

(1) **Extract the valid regions of the image.** To obtain the geographic coordinates of SAR images, orthorectification is first applied. However, invalid pixels in the orthorectified images may reduce the accuracy of seamless mosaicking [14], making it necessary to extract valid image regions. In practice, valid regions correspond to non-zero pixels, while invalid regions are zero-valued background pixels, typically surrounding the valid regions. For clarity, non-zero pixels adjacent to background pixels are referred to as *contour pixels*. Let the coordinates of the initial contour pixel be $\left(x_{0},y_{0}\right)$. By traversing its eight neighboring directions, the next contour pixel $\left(x_{1},y_{1}\right)$ is located. This process is repeated for $\left(x_{1},y_{1}\right)$, then $\left(x_{2},y_{2}\right)$, and so forth, until $\left(x_{n},y_{n}\right)$ coincides with $\left(x_{0},y_{0}\right)$. In this way, all contour pixels are extracted, forming a closed boundary that delineates the valid region. The valid regions of an orthorectified image are illustrated in Fig 6.
(2) **Extraction of Image Overlapping Regions.** The detection of the optimal seamline within the overlapping regions of adjacent images not only ensures the rationality and feasibility of image mosaicking, but also effectively reduces the search space and improves computational efficiency. The valid regions of SAR satellite images are generally represented as simple polygons. The overlapping valid areas between adjacent orthorectified images can thus be obtained by computing the intersections of these polygons using the Weiler–Atherton algorithm [44]. As illustrated in Fig 7, let the two intersecting polygons be *P* and *Q*, with vertices $p_{n}$ and $q_{n}$ (*n* = 1,2,3,4), respectively. Suppose the edge $\overset{―}{\left(p_{2},p_{3}\right)}$ intersects with $\overset{―}{\left(q_{1},q_{2}\right)}$ at point *A*, and $\overset{―}{\left(p_{3},p_{4}\right)}$ intersects with $\overset{―}{\left(q_{1},q_{4}\right)}$ at point *B*. To clip polygon *Q* with polygon *P*, the intersection point *A* is defined as an *entry point*, and *B* as an *exit point*. After inserting these intersection points, the vertex sequences of polygons *P* and *Q* become $\left(p_{1},p_{4},B,p_{3},A,p_{2}\right)$ and $\left(q_{1},B,q_{4},q_{3},q_{2},A\right)$, respectively. Starting from the entry point *A*, the clipped polygon *Q* is traversed in the counterclockwise direction to obtain the sequence (*A*,*q*_1_). Upon reaching the exit point *B*, the traversal continues along polygon *P* in the counterclockwise direction to form the sequence $\left(A,q_{1},B,p_{3}\right)$. The process terminates when the sequence returns to the entry point *A*. Connecting the points in this sequence forms a closed polygon that represents the overlapping region between polygons *P* and *Q*.
(3) **Skeleton Extraction.** The skeleton or medial axis of a polygon can be derived using the *Grassfire model*, which simulates the process of “igniting” all edges simultaneously, allowing the fire fronts to propagate inward at a uniform speed and connecting the points where the fire fronts meet and extinguishes [45]. As illustrated in Fig 8, let the polygon $P_{1}P_{2}P_{3}P_{4}$ represent the overlapping region, where *P*_1_, *P*_2_, *P*_3_, and *P*_4_ denote the polygon vertices. Construct the angle bisectors $\overset{―}{\left(P_{1}M_{1}\right)}$, $\overset{―}{\left(P_{2}M_{1}\right)}$, $\overset{―}{\left(P_{3}M_{2}\right)}$, and $\overset{―}{\left(P_{4}M_{2}\right)}$. The intersection of $\overset{―}{\left(P_{1}M_{1}\right)}$ and $\overset{―}{\left(P_{2}M_{1}\right)}$ is denoted as *M*_1_, while that of $\overset{―}{\left(P_{3}M_{2}\right)}$ and $\overset{―}{\left(P_{4}M_{2}\right)}$ is denoted as *M*_2_. Among these vertices, *P*_1_ and *P*_3_ correspond to the intersection points *A* and *B* of adjacent polygons, respectively (see Fig 10). By connecting point *P*_1_(*A*) to *M*_1_ and point *P*_3_(*B*) to *M*_2_, the resulting line $\overset{―}{\left(P_{1}M_{1}M_{2}P_{3}\right)}$ represents the skeleton of the overlapping region between adjacent images [13].

**Fig 9:**
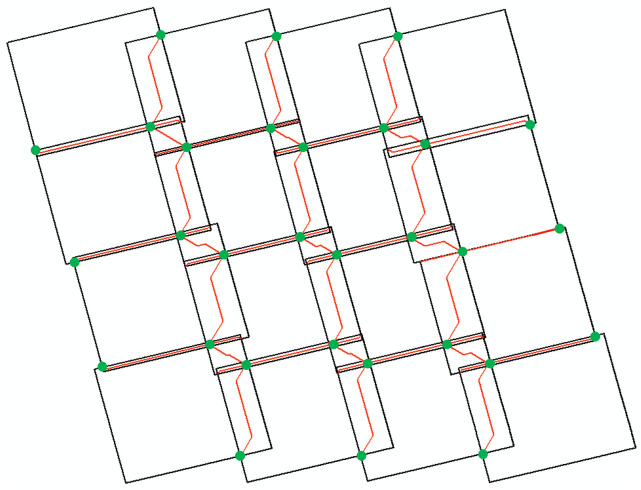
Initial seamline network among multiple SAR images.

For the multiple SAR images in the study area, a skeleton line can be extracted from each overlapping region between adjacent image pairs. These skeleton lines collectively form the initial seamline network, with their intersections serving as network nodes. Fig 9 presents the initial seamline network constructed from 16 overlapping ALOS SAR images. In this figure, black polygons denote the effective image regions, red line represent the initial seamlines generated by the AVDO, and green dots indicate the network nodes. Internal nodes correspond to intersections of skeleton lines, while peripheral nodes correspond to intersections at the boundaries of effective image regions.

#### 2.3.2 Extraction of optimal seamlines.

The initial seamlines generated by the AVDO are determined solely by the geometric shape of the overlapping SAR image regions, without considering the actual ground features contained within. As a result, these seamlines may traverse intact features, leading to suboptimal results. To overcome this limitation, the normalized cross-correlation (NCC) [46] is adopted as a cost function, and shortest-path algorithm is applied to extract the optimal seamlines from the candidate set. The procedure consists of the following steps:

**Algorithm 1 Optimal Seamline Extraction**



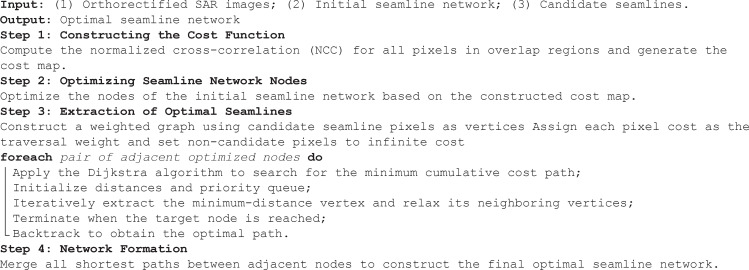

(1) **Constructing the Cost Function.** Ideally, overlapping SAR images should exhibit identical intensity values. In practice, however, seasonal variations, signal transmission errors, and other factors often cause mismatches in overlapping regions. The optimal seamline should pass through pixels with minimal mismatch. The mismatch at each pixel is translated into a cost. The greater the mismatch, the higher the cost for the seamline passing through that pixel. Conversely, lower mismatch implies lower cost, making it preferable.

Disparity maps are commonly employed to quantify this cost because of their simplicity and efficiency [47]. A disparity map records the intensity differences between corresponding points in overlapping images, but its accuracy is highly dependent on registration quality. Inaccurate registration may yield erroneous disparity maps, resulting in seamlines that traverse mismatched areas. To mitigate sensitivity to registration errors, neighborhood-based NCC is used to measure the cost associated with each pixel. For any two overlapping pixels *p*, *q* at location (*x*, *y*), the NCC is computed as:


$${\text{NCC}}_{pq}(x,y)=\frac{\sum_{i=x-w}^{x+w}\sum_{j=y-w}^{y+w}(f_{p}(i,j)-{\bar{f}}_{p}(x,y))(f_{q}(i,j)-{\bar{f}}_{q}(x,y))}{\sqrt{\sum_{i=x-w}^{x+w}\sum_{j=y-w}^{y+w}(f_{p}(i,j)-{\bar{f}}_{p}(x,y){)}^{2}\sum_{i=x-w}^{x+w}\sum_{j=y-w}^{y+w}(f_{q}(i,j)-{\bar{f}}_{q}(x,y){)}^{2}}}$$ (14)

where *w* is the window size (set to 5×5 in this study), $f_{p}(i,j)$ and $f_{q}(i,j)$ denote the intensity values of pixel (*i*,*j*) in images *p* and *q*, respectively, and ${\bar{f}}_{p}(x,y)$ and ${\bar{f}}_{q}(x,y)$ are the mean intensities within the window.

For locations with multiple overlapping images, NCC values are calculated for all overlapping pixel pairs, and the maximum value is retained as the final index at that location:


$$\text{NCC}(x,y)=\max\{{\text{NCC}}_{pq}(x,y)\},\;(p,q=1,2,\ldots ,s;\;p\neq q)$$ (15)

The NCC value ranges from [−1,1] and is converted to the cost function ranging [0, 1] via:


$$\text{cost}(x,y)=\frac{1-\text{NCC}(x,y)}{2}$$ (16)

where cost(*x*, *y*) denotes the cost of the seamline passing through location (*x*, *y*).

**Fig 10:**
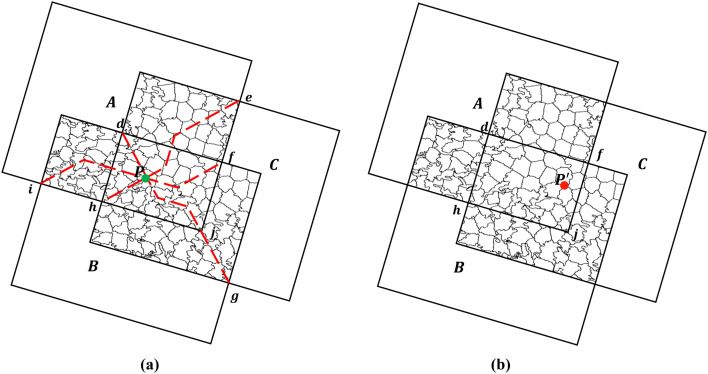
Optimization of Network Nodes. **(a)** Intersection points of skeleton lines in overlapping regions. **(b)** Optimized network nodes.

(2) **Optimizing Seamline Network Nodes.** The nodes of the initial seamline network fall into two categories: (1) intersections of skeleton lines, located within the network interior; and (2) intersections of effective image regions, located at the network boundary. Skeleton line intersections may coincide with high-mismatch pixels, which would increase the total cost if directly used as network nodes. To avoid this and to ensure alignment with the candidate seamlines extracted in section 2.2, internal nodes are relocated to positions with the lowest mismatch along the candidate lines. Specifically, for a skeleton line intersection within an overlapping region, the cost is computed along all candidate seamlines in that region, and the position with the minimum cost is chosen as the updated node. As illustrated in Fig 10, $\overset{―}{dg}$ represents the skeleton line of the overlap between images B and C, $\overset{―}{if}$ between A and B, and $\overset{―}{eh}$ between A and C. The candidate seamlines are curves within the overlapping region. The intersection of the three skeleton lines is *P*, located in the common overlap *dfjh* of the three images. The cost for candidate seamline positions is computed as:

$$\text{cost}(x,y{)}_{\left(A,B\right)}=\frac{1-\text{NCC}(x,y{)}_{\left(A,B\right)}}{2}$$ (17)

$$\text{cost}(x,y{)}_{\left(A,C\right)}=\frac{1-\text{NCC}(x,y{)}_{\left(A,C\right)}}{2}$$ (18)

$$\text{cost}(x,y{)}_{\left(B,C\right)}=\frac{1-\text{NCC}(x,y{)}_{\left(B,C\right)}}{2}$$ (19)

The final cost at location (*x*, *y*) is:


$$\text{cost}(x,y)=\min\{\text{cost}(x,y{)}_{\left(A,B\right)},\text{cost}(x,y{)}_{\left(A,C\right)},\text{cost}(x,y{)}_{\left(B,C\right)}\}$$ (20)

The new network node $P^{\prime }$ is then determined as the pixel with the minimum cost among all candidate seamline positions:


$$P^{\prime }=\min\{\text{cost}(x_{i},y_{i})\}$$ (21)

(3) **Extraction of Optimal Seamlines.** After updating the network nodes, the optimal seamline between adjacent nodes is extracted from the set of candidate seamlines. The objective is to minimize the cumulative cost of all pixels along the path. To ensure that the seamline adheres closely to ground-object boundaries and traverses only candidate seamline pixels, the cost of pixels outside the candidate lines is set to infinity. Accordingly, the cost in the overlapping region is defined as:

$$\text{cost}(x,y)=\left\{ \begin{array}{cc} \frac{1-\text{NCC}(x,y)}{2}, & (x,y)\in \text{candidate seamline}\\ \infty , & (x,y)\notin \text{candidate seamline} \end{array}\right.$$ (22)

Identifying the optimal seamline between adjacent nodes is therefore framed as a path-planning problem [48]. Based on the cost function in Eq (22), the optimal seamline is determined using Dijkstra’s algorithm. Collectively, all extracted seamlines constitute the optimal seamline network.

## 3 Results and discussions

### 3.1 Method validation

To validate the effectiveness of the proposed method, two overlapping images were processed following the procedures described in Section 2, including superpixel segmentation, region merging, and optimal seamline extraction. The experimental results are presented as follows.

**Fig 11:**
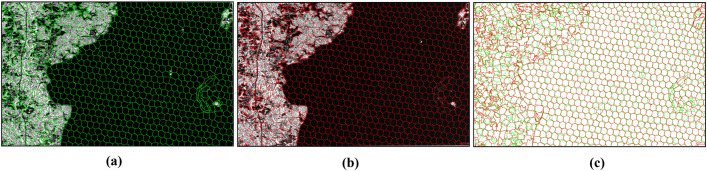
Segmentation results of the PB-SLIC algorithm. **(a)** Segmentation result of image **A. (b)** Segmentation result of image **B. (c)** Overlay of the two segmentation results.

**Fig 12:**
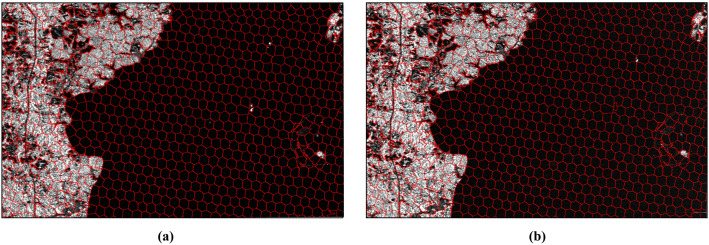
Segmentation results of the proposed algorithm. **(a)** Segmentation result of image **A. (b)** Segmentation result of image **B.**

First, the PBS-SLIC algorithm and the proposed method were applied to perform superpixel segmentation on the images. The segmentation results are shown in Figs 11 and 12. It can be observed that the PBS-SLIC algorithm accurately captures object boundaries in both images and achieves satisfactory segmentation performance. However, the two segmentation results do not coincide in the overlapping region, exhibiting evident inconsistency. In contrast, the proposed method, while inheriting the favorable properties of the original algorithm, successfully achieves consistent segmentation for overlapping images A and B. Therefore, the proposed method effectively aggregates pixels with similar intensity characteristics into superpixels and provides consistent and accurate superpixel boundaries for different images within the overlapping region.

**Fig 13:**
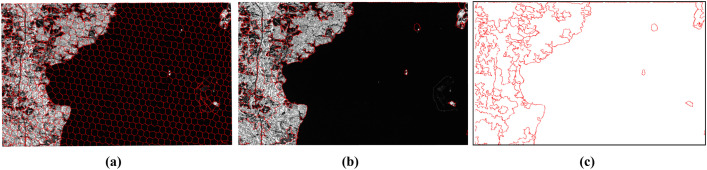
Results of Superpixel Merging and Candidate Seamline Extraction. **(a)** Before merging. **(b)** After merging. **(c)** Candidate seamlines.

Subsequently, region merging was performed based on the segmentation results of the proposed method. Fig 13 illustrates a comparison before and after superpixel merging. Owing to the inherent characteristics of superpixel segmentation algorithms, the proposed method still exhibits over-segmentation in certain areas. For example, the water body on the right side of the image in Fig 13(a) is divided into multiple superpixels. If these segmentation boundaries were directly used as candidate seamlines, the efficiency and quality of subsequent optimal seamline detection would inevitably be reduced. After integrating multiple similarity measures to merge adjacent superpixels, the water body on the right side is reassembled into a single region, as shown in Fig 13(b). This demonstrates that the region merging strategy effectively recombines homogeneous pixels that were previously over-segmented into complete objects. The superpixel boundaries after region merging are then taken as candidate seamlines, as illustrated in Fig 13(c).

**Fig 14:**
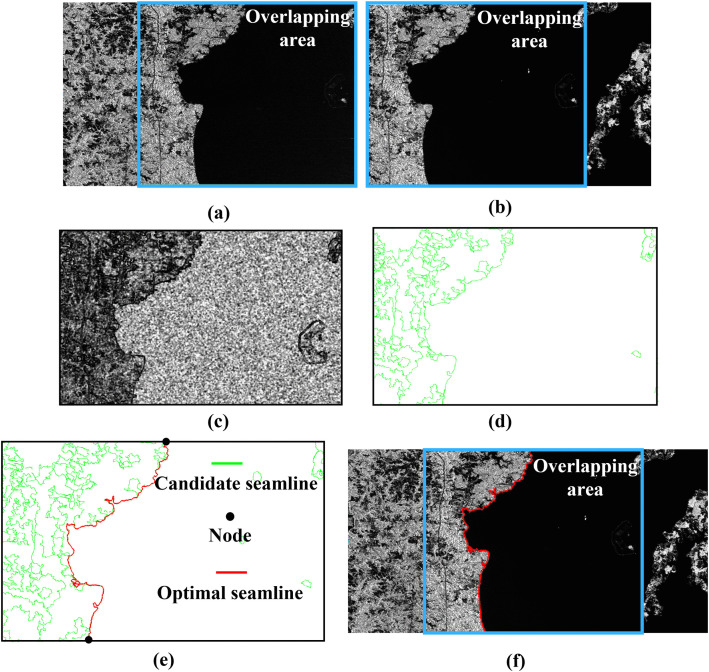
Extraction of the Optimal Seamline. **(a)** Left image, with the blue area representing the overlapping region. **(b)** Right image, with the blue area representing the overlapping region. **(c)** Cost matrix. **(d)** Candidate seamline. **(e)** Refined result: green curves indicate candidate seamlines, black dots represent network nodes, and red curves indicate the optimal seamline. **(f)** Illustration of the optimal seamline overlaid on the images to be mosaicked: red curves show the optimal seamline, and the blue area represents the overlapping region.

Finally, the optimal seamline was extracted from the candidate seamlines based on the constructed cost function and the shortest-path algorithm, as shown in Fig 14. The resulting seamline corresponds to the boundary between the water body and the surrounding land, indicating that the proposed method is capable of accurately aligning the seamline with actual object boundaries. By connecting all optimal seamlines extracted from the overlapping regions, a complete optimal seamline network can be formed.

These results demonstrate that the proposed segmentation algorithm effectively conforms to object boundaries while ensuring consistency within overlapping regions. With the introduction of the superpixel merging strategy, fragmented objects caused by over-segmentation are reorganized into complete entities, thereby generating candidate seamlines that both adhere closely to object boundaries and preserve object integrity. On this basis, the integration of the constructed cost function and the shortest-path algorithm enables the extraction of optimal seamlines from the candidate set, effectively avoiding the problem of seamlines crossing intact objects.

### 3.2 Comparative evaluation of optimal seamline networks

#### 3.2.1 Experimental dataset.

**Fig 15:**
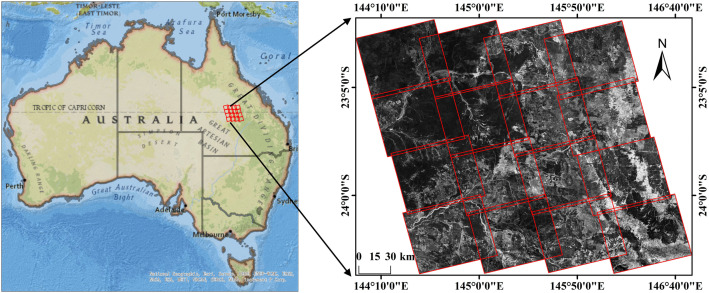
Data Coverage.

Some ALOS PALSAR L-band SAR images were selected for testing. The acquisition dates of the images range from 2007 to 2010, and both HH and HV polarization modes are included. Each image contains approximately 3000 × 2500 pixels with a spatial resolution of 12.5 m. As illustrated in Fig 15, the selected images are located in eastern Australia, covering an area between 22.50°–24.66° S latitude and 143.95°–146.80° E longitude, with a total extent of approximately 42,000 km². The terrain is primarily composed of low hills and alluvial plains, with diverse land cover including grasslands, sparse vegetation, cropland, and localized forests. Detailed dataset information is provided in Table 1.

**Table 1:** Data Information.

Image ID	Polarization	Acquisition Date	Image ID	Polarization	Acquisition Date
ALPSRP081756720	HH	2007-08-07	ALPSRP184156690	HH	2009-07-09
ALPSRP121286700	HV	2008-05-04	ALPSRP190866700	HV	2009-08-24
ALPSRP121286720	HH	2008-05-04	ALPSRP197576710	HH	2009-10-09
ALPSRP122016690	HV	2008-05-09	ALPSRP204286720	HV	2009-11-24
ALPSRP122016710	HH	2008-05-09	ALPSRP226896700	HH	2010-04-28
ALPSRP127996690	HV	2008-06-19	ALPSRP226896720	HV	2010-04-28
ALPSRP134706710	HH	2008-08-04	ALPSRP240316690	HH	2010-07-29
ALPSRP135436700	HV	2008-08-09	ALPSRP240316710	HV	2010-07-29

Prior to seamline detection, the data underwent orthorectification and radiometric consistency correction. Orthorectification, performed using DEM data, eliminates distortions caused by terrain relief—such as displacement, shadows, and occlusions—and projects the images into a geographic coordinate system. Appropriate radiometric correction models were applied to reduce differences among images caused by variations in incidence angle, acquisition time, and other factors, thereby preventing degradation of seam-line quality due to radiometric inconsistencies.

#### 3.2.2 Experimental results.

**Fig 16:**
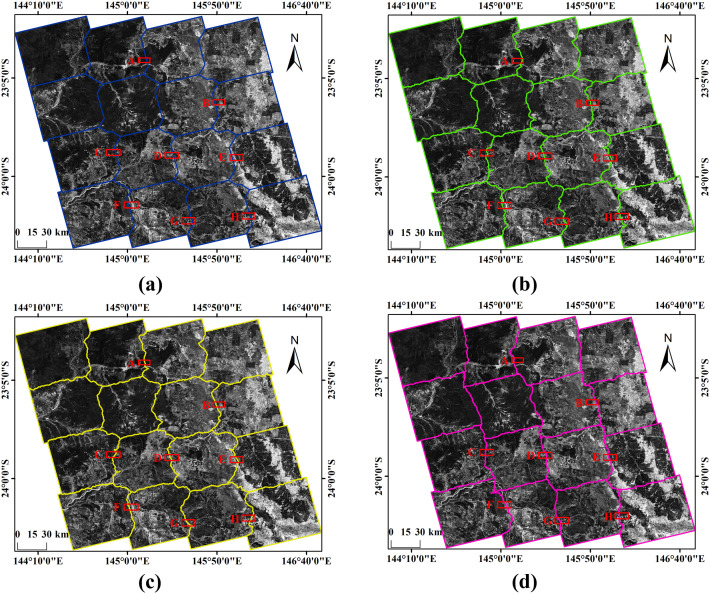
Overall Results. **(a)** Initial seamline network. **(b)** Result of the method proposed by Pan et al. **(c)** Result of the method proposed by Wang et al. **(d)** Result of the proposed method in this study.

**Fig 17:**
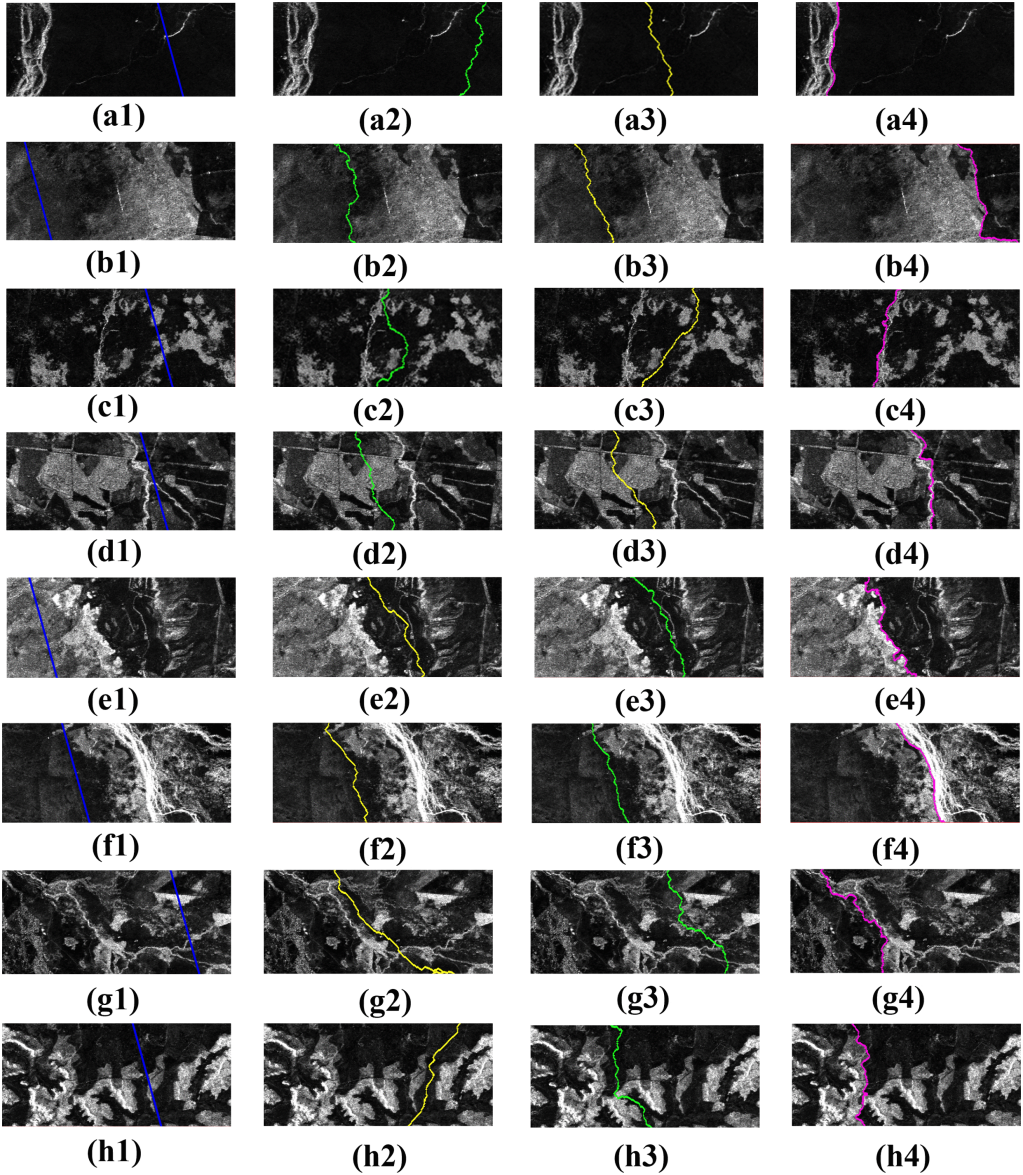
Local Area Enlargements. (a1–h1) Enlarged local details of the initial seamline network, corresponding to the red boxes in Fig 16(a). (a2–h2) Enlarged local details of the results from Pan et al.’s method, corresponding to the red boxes in Fig 16(b). (a3–h3) Enlarged local details of the results from Wang et al.’s method, corresponding to the red boxes in Fig 16(c). (a4–h4) Enlarged local details of the results from the proposed method in this study, corresponding to the red boxes in Fig 16(d).

To evaluate the effectiveness and reliability of the proposed method, comparisons were made with Pan et al.’s seamline extraction method based on AVDO [14] and Wang et al.’s spatial ant colony optimization-based seamline extraction method [19]. Both approaches first generate an initial seamline network automatically from a VD and then refine it using the shortest-path algorithm and ant colony optimization, respectively, to obtain the final seamline network. The overall experimental results are presented in Fig 16, while Fig 17 provides a zoomed-in view of a local region.

#### 3.2.3 Visual analysis.

In the experimental results, the green seamlines correspond to the overlapping area–based Voronoi diagram method proposed by Pan et al., the yellow seamlines represent the spatial ant colony optimization–based method proposed by Wang et al., and the pink seamlines denote the method proposed in this study. Overall, the seamline networks generated by Pan et al.’s and Wang et al.’s methods exhibit similar spatial distributions. Both approaches construct the cost function primarily based on intensity and texture differences and search for optimal paths within low-difference regions. However, due to the lack of explicit structural constraints from ground features, their seamlines often fail to follow actual feature boundaries and may traverse intact objects. In contrast, the proposed method constrains the search space using candidate seamlines (i.e., feature boundaries), resulting in optimal seamlines that better conform to object structures, significantly reducing the probability of crossing prominent features and yielding improved overall visual quality.

To further evaluate the visual performance of the three methods, seamline extraction results were compared across eight local areas (Areas A–H). In flat regions A–D, where terrain undulation is minimal and geometric distortions are limited, texture and intensity differences between adjacent images are relatively weak. In such homogeneous areas, the methods of Pan et al. and Wang et al. are capable of identifying paths with small radiometric variations, and no obvious brightness discontinuities appear in the mosaicking results. Nevertheless, because the optimization relies solely on pixel similarity, some seamlines directly traverse regular farmlands or intact parcels, dividing a single object into parts derived from different images and thereby weakening structural integrity and continuity. By contrast, the proposed method demonstrates stronger structural consistency in these regions: the seamlines closely follow road edges or farmland boundaries, preserving the integrity of regular parcels and achieving a balance between radiometric consistency and structural completeness.

In mountainous regions E–H, the differences among the methods become more pronounced. Influenced by complex terrain and parallax effects, these areas exhibit substantial variations in intensity and texture. Although the methods of Pan et al. and Wang et al. can avoid high-cost regions, their seamlines often meander within locally low-difference areas without adhering to true terrain boundaries such as ridgelines, valley lines, or zones of abrupt topographic change. In some cases, seamlines even pass through continuous mountain shadow regions, resulting in structural discontinuities and visual artifacts. In comparison, guided by the feature-boundary–constrained mechanism, the proposed method preferentially aligns seamlines along ridgelines or terrain transition zones, effectively avoiding continuous mountainous areas and producing visually more natural mosaicking results.

In summary, although the methods of Pan et al. and Wang et al. can generate relatively smooth paths in areas with small radiometric differences, the absence of explicit structural constraints leads to seamlines that cross intact objects in multiple regions, which may adversely affect subsequent image fusion and fine-scale applications. Benefiting from the precise delineation of feature boundaries provided by candidate seamlines, the proposed method consistently produces seamlines that adhere closely to object structures even in complex scenarios. It achieves superior visual continuity and obstacle-avoidance capability in both flat and mountainous regions, thereby demonstrating its effectiveness and robustness in challenging environments.

#### 3.2.4 Statistical analysis.

To quantitatively evaluate performance, two metrics were employed. The first metric is visual inconsistency, which measures the discontinuity in radiometric intensity across the seamline, thereby reflecting perceptual inconsistency introduced during mosaicking [46]. This metric is commonly defined as the sum of the largest N costs along the seamline. To avoid the subjectivity associated with selecting N, this study instead adopts the root mean square error (RMSE) of all pixel differences along the seamline:


$$RMSE=\sqrt{\frac{\sum_{i=1}^{n}D_{p,q}^{2}(x_{i},y_{i})}{n}}$$ (23)

$$D_{p,q}(x,y)=\max\left|f_{p}(x,y)-f_{q}(x,y)\right|$$ (24)

where $D_{p,q}(x,y)$ is the pixel intensity difference at location (*x*, *y*) along the seamline, and p,q=1,2,…,n with p≠q.

The second metric is search time, defined as the time required to identify the optimal seamline, which provides a measure of computational efficiency. The results of both metrics are summarized in Table 2.

**Table 2:** Quantitative Metric Results.

Metric	Pan et al., 2014	Wang et al., 2022	Proposed Method
Visual Inconsistency	0.749	0.792	0.613
Search Time (s)	212.055	181.160	50.709

Overall, the differences in visual inconsistency among the three methods are relatively small. The visual inconsistency values of the methods proposed by Pan et al., Wang et al., and the present study are 0.749, 0.792, and 0.613, respectively. Compared with the approaches of Pan et al. and Wang et al., the proposed method reduces visual inconsistency by 18.16% and 22.60%, respectively. These results indicate that the proposed method more effectively suppresses radiometric discontinuities and texture inconsistencies within the mosaicked regions, thereby achieving the best visual consistency among the three methods.

In terms of search efficiency, the differences are more pronounced. The search times of Pan et al.’s and Wang et al.’s methods are 212.055 s and 181.160 s, respectively. Wang et al.’s method demonstrates an improvement in efficiency over that of Pan et al., mainly because Pan et al.’s approach searches eight subregions within the overlap area, whereas Wang et al.’s method reduces the search to five subregions. By contrast, the proposed method requires only 50.709 s, representing reductions of 76.09% and 72.01% compared with the former two methods, respectively, and thus significantly improving computational efficiency.

The fundamental reason for this substantial difference lies in the search strategy. The first two methods perform global or semi-global searches over all pixels within the overlapping region, and their computational cost increases approximately linearly with the size of the overlap area. In contrast, the proposed method constructs candidate seamlines in advance and restricts the search space to a subset of candidate pixels. Only those pixels that are likely to form the final seamline are evaluated, which markedly reduces redundant computations and unnecessary searches. Consequently, the proposed method requires the least processing time and achieves the highest overall efficiency.

## 4 Conclusions

This study presents an optimal seamline detection method for SAR image mosaicking, guided by superpixel segmentation and region merging, aiming to improve both seamline quality and computational efficiency in large-area applications. The approach explicitly accounts for the characteristics of SAR image by enhancing the PB-SLIC algorithm, enabling the direct generation of accurate and consistent superpixels within overlapping multiple images. Building upon this segmentation, superpixels are effectively merged using a combination of Bhattacharyya distance, texture descriptors, and boundary length constraints, thereby mitigating the adverse effects of over-segmentation and producing candidate seamlines that precisely follow feature boundaries while preserving structural integrity. Subsequently, these candidate seamlines are refined through a cost function and shortest-path optimization, resulting in the construction of the final optimal seamline network. Experimental evaluations confirm that the proposed method successfully avoids crossing intact features and achieves superior visual consistency, while significantly reducing computation time compared with existing approaches.

However, the proposed method performs seamline searching within the overlapping regions of adjacent images. When the overlap is excessively narrow or contains large-scale continuous features, the seamline may have limited flexibility to effectively bypass such objects, and consequently may still traverse intact features. In addition, the extraction of candidate seamlines requires superpixel segmentation and subsequent region merging, which increases the complexity of the processing workflow.

Future work will therefore focus on further improving SAR image segmentation, with the objective of eliminating the region-merging step and directly obtaining satisfactory segmentation results. Such refinement is expected to simplify the overall framework of the proposed algorithm while maintaining its effectiveness in optimal seamline detection. Furthermore, more statistical analysis will be introduced in future studies to provide a more thorough and objective assessment of seamline quality and mosaicking performance.

## Supporting information

S1 Dataset The orthorectified SAR image dataset used in this study. (RAR)

S2 Dataset The orthorectified SAR image dataset used in this study. (RAR)

S3 Dataset The orthorectified SAR image dataset used in this study. (RAR)

S4 Code The code for processing the SAR images dataset. (ZIP)
