# Supplementary figures and images for: Optimal seamline detection for SAR image mosaicking guided by superpixel segmentation and region merging
Source: PLoS One. 2026 May 8;21(5):e0348842. doi: 10.1371/journal.pone.0348842 (PMC13155582; doi:10.1371/journal.pone.0348842)

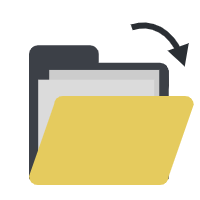

Supplement: S4 Code — (ZIP) [file pone.0348842.s004.zip › Code/src/res/icons/open.png]

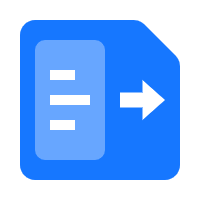

Supplement: S4 Code — (ZIP) [file pone.0348842.s004.zip › Code/src/res/icons/out.png]

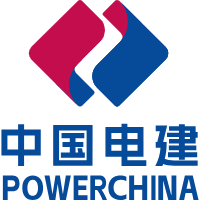

Supplement: S4 Code — (ZIP) [file pone.0348842.s004.zip › Code/src/res/icons/powerchina.png]

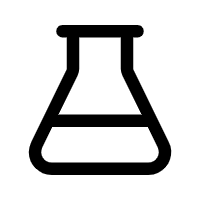

Supplement: S4 Code — (ZIP) [file pone.0348842.s004.zip › Code/src/res/icons/window.png]
